# 

**Published:** 1904-01-09

**Authors:** 


### Publications.

Just Published. 8vo. with Photographic reproductions, 3/- net.

**THE PHYSIOGNOMY OF MENTAL DISEASES AND DEGENERACY.** By JAMES SHAW, M.D., formerly Med. Supt. and Co-Licensee Haydock Lodge Asylum. Reprinted, with alterations and additions, from various articles in the "Medical Annual."

Just Published. Crown 8vo. 1/6 net.

**A POCKET BOOK OF CLINICAL METHODS.** By CHAS. H. MELLAND, M.D.Lond., M.R.C.P., Phys. Ancoats Hosp.

THIRD EDITION. 12th Thousand. Small 8vo. Illustrated with 200 Original Drawings, 2/6. Lantern Slides of the Illustrations, 1/- and 1/6 each.

**"FIRST AID" TO THE NJURED AND SICK:** An Advanced Ambulance Handbook. By F. J. WARWICK, B.A., M.B., and A. C. TUNSTALL, M.D., F.R.C.S.

THIRD EDITION. 2/- each, or 27/6 the Set of 16 Sheets, or mounted on linen, 45/- With nickel head.

**(Adopted by the War Office, Admiralty, and London School Board.)**

**LARGE SHEET "FIRST AID" DIAGRAMS:** Being Enlargements of the Illustrations in the above work on sheets 26 in. by 40 in., suitable for lectures and classes.

### MEDICAL ACCOUNTS.

Now is the time to arrange for this troublesome business, and

**WRIGHT'S THREE-BOOK SYSTEM,**
**WRIGHT'S SINGLE-BOOK SYSTEM,**
(11th Edition)
**WRIGHT'S OBSTETRIC RECORDS,**
**WRIGHT'S VISITING LISTS, 1904,**
**WRIGHT'S CHARTS of all kinds,**
(Nearly 400,000 of which have been sold)
**WRIGHT'S CHART HOLDERS,**
(In use all over the world)
**WRIGHT'S CERTIFICATE BOOKS,**
**PROFESSIONAL ACCOUNT FORMS,**
**LETTER HEADINGS, and**
**DISPENSING LABELS.**

Are most of them better than the ordinary run of these things, and their use saves time and trouble.

PLEASE SEND FOR FREE SAMPLES.

Bristol: JOHN WRIGHT & CO. London: SIMPKIN & CO., Ltd.

---

FOURTH EDITION. Plates contain 93 Coloured and Stereoscopic Figures, 209 Illustrations in the Text, 17/6 net, including Stereoscope.

By P. WATSON WILLIAMS, M.D.(Lond.),
Physician in Charge of Throat Department,
Bristol Royal Infirmary, &c., &c.

### DISEASES OF THE NOSE, PHARYNX, AND LARYNX.

WITH A SECTION ON THE EXAMINATION OF

**THE EAR.**

**Press Notices.**—"We know of no better practical guide."—*Practitioner.* "Certain to receive the recommendation of all teachers."—*Brit. Med. Journ.* "We can strongly recommend the book."—*Lancet.* "Excellent......The features of the work—completeness, clear description, and practical utility—are absolutely maintained in the new edition.—(Transn.)." *Internat. Centr. f. Laryngologie.*

Bristol: J. WRIGHT & CO. London: SIMPKIN & CO.

JUST ISSUED. EIGHTH EDITION. Crown 8vo, 10s. 6d.

### PHARMACY, MATERIA MEDICA AND THERAPEUTICS

Is now rewritten and printed in large type, and is made a companion to the following volume. It contains an account of the action **of every drug in the B.P.,** as well as a section upon all the newest drugs, with an Introduction to the Art of Dispensing. With woodcuts, prescriptions, &c

Also FOURTH EDITION. 1055 pages, 8vo. 16s.

### DICTIONARY OF TREATMENT.

Containing a Revised and up-to-date Article upon every Diseased Condition, whether Medical, Surgical, Gynæcological, or Ophthalmological, as well as Articles upon Poisoning, Drowning, and the various Surgical Emergencies and Accidents, arranged for Rapid Reference.

By W. WHITLA, M.A., M.D., Professor of Materia Medica, Queen's College, Belfast; Senior Physician to the Royal Victoria Hospital, &c.

The Publisher will supply both volumes, carriage paid, for 21/-

London: HENRY RENSHAW, 356 Strand.

Crown 8vo. Illustrated. Price 2s. 6d. net.

### ANÆSTHETICS. By J. BLUMFELD,

M.D.Cantab., Senior Anæsthetist to St. George's Hospital; Anæsthetist to the Grosvenor Hospital for Women and Children, London, and to the National Dental and Metropolitan Hospitals.

*Lancet.*—"....In writing this handbook the author has been quite successful." *Australasian Medical Gazette.*—"We can strongly commend this book." *Medical Chronicle.*—"The book is one which we can heartily recommend to senior students and practitioners."

London: BAILLIÈRE, TINDALL & COX, 8 Henrietta St., Covent Garden.

NOW READY. Demy 8vo. Price 5/- net.

### ASTHMA IN RELATION TO THE NOSE.

*WITH NOTES OF 402 CASES.*

By ALEXANDER FRANCIS, M.B., B.C.Cantab.

London: ADLARD & SON, Bartholomew Close, E.C.

Second Edition. Crown 8vo., price 3/6 net.

With original "Plea—1900—for the State Registration of Nurses."

### MOCK NURSES OF THE LATEST FASHION.

By FREDERICK J. GANT, F.R.C.S.,
Consulting Surgeon to the Royal Free Hospital.

BAILLIÈRE, TINDALL & COX,
Henrietta Street, Covent Garden, London.

### NOTICE TO CORRESPONDENTS.

All MSS., Letters, Books for Review, and other matters intended for the Editor should be addressed The Editor, "The Hospital," The Hospital Buildings, 28 & 29 Southampton Street, Strand, W.C.

The Editor cannot undertake to return rejected MS., even when accompanied by stamped directed envelope.

**Notice to Advertisers and Subscribers.**

All Advertisements, Orders for Copies of The Hospital, and Business Communications should be addressed to **THE MANAGER (Not to the Editor),** The Hospital, 28 & 29 Southampton Street, Strand, London, W.C.

The Cost of Subscription, post free, is as follows:—Per week, 3½d.; per quarter, 3s. 9d.; per half-year, 7s. 6d.; per annum, 15s. Foreign and Colonial:—Per annum, 19s. 6d., payable, in all cases, in advance.

**NOTICE.—Vol. XXXIV. of "The Hospital" (April 1903 to September 1903), in handsome cloth binding, is now on sale at the office of this Journal, price 10s. 6d. Cases for binding the Numbers contained in this Volume 2s. Index to Vol. XXXIV., 2½d., post free.**

**The Monthly part for January is now ready. Price 1s., by post, 1s. 3d.**

**THE HANDBOOK OF THE OPEN-AIR TREATMENT**

By Dr. CHARLES REINHARDT

(Visiting Physician to Hailey Sanatorium, Wallingford, and Stourfield Park Sanatorium, Bournemouth),

Contains Views, Descriptions, and Information respecting 30 Sanatoria in Great Britain.

Price: Cloth, 2/6; Paper, 1/- (postage 4d.)

The "HEALTH RESORT" Publishing Office, 379 Strand, London, W.C.

### THE STUDENT OF MEDICINE.

### APPOINTMENTS.

**J. H. Blayney**, L.F.P.S.Glasg., Certifying Factory Surgeon for the Middleton District, Lancashire. **A. J. Boyd**, B.A., M.D.Dub., Honorary Assistant Medical Officer Hertford General Infirmary. **R. E. Crosse**, M.R.C.S.Eng., L.R.C.P.Lond., Medical Officer and Public Vaccinator of the Mitford and Launditch Union Workhouse. **H. H. Folker**, M.R.C.S., L.R.C.P., Certifying Factory Surgeon for the Hanley District, Staffordshire. **R. Garbutt**, L.R.C.P.&S.Edin., Medical Officer for the Wolsingham District of the Weardale Union. **F. Isherwood**, L.R.C.P.&S. Edin., L.F.P.S.Glasg., Certifying Factory Surgeon for the Cockermouth District, Cumberland. **H. Horsman McNabb**, M.D., Assistant Honorary Surgeon to the Manchester Eye Hospital. **John Douglas Stanley**, M.D.Edin., M.R.C.P.Lond., Honorary Physician to the Birmingham Children's Hospital. **H. N. Turner**, M.B., Certifying Factory Surgeon for the Castle Bytham District, Lincolnshire. **Henry Wade**, M.B., F.R.C.S.Edin., Conservator of the Museum of the Royal College of Surgeons, Edinburgh. **J. D. Walker**, M.B., C.M.Aberd., Certifying Factory Surgeon for the Shaftesbury District, Dorsetshire. **C. Gordon Watson**, F.R.C.S.Eng., Assistant Surgeon to the Metropolitan Hospital. **S. R. Williams**, M.R.C.S., L.R.C.P., Clinical Assistant to the Chelsea Hospital for Women. **H. H. Williamson**, M.R.C.S., L.S.A., Certifying Factory Surgeon for the Ludgershall District, Wiltshire.

**"The Hospital" Institutional Library.**

**"Hospitals and Asylums of the World." 4 Vols., with a Portfolio of Plans, complete £12 12s.**

**"Burdett's Hospitals and Charities, 1903." 5s. net.**

**"Burdett's Official Nursing Directory, 1903." 3s. net.**

**"Cottage Hospitals, General, Fever, and Convalescent." 10s. 6d.**

**"Uniform System of Accounts for Hospitals and Public Institutions." New Edition. 4s. net.**

**"Hospital Expenditure: The Commissariat." 2s. 6d.**

**"A Legal Handbook for the Use of Hospital Authorities." 2s. 6d.**

**"The Cottage Hospital Case Book or Register of Patients." 10s. and 12s. 6d.**

**All these are published by the SCIENTIFIC PRESS, Ltd., and may be obtained through any bookseller, or direct from the publishers, 28 and 29 Southampton Street, Strand, London, W.C.**

*Just Published.*

### THE EXAMINATION OF THE URINE.

By J. K. WATSON, M.D Edin., M.B., C.M.

*Author of "A Handbook for Nurses."*

Price **1/-** net; post free **1/1.**

*Of all Booksellers, or of*

THE SCIENTIFIC PRESS, LTD, 28 & 29 Southampton Street, Strand, London, W.C.

THE

### BURDETT SERIES

OF

### POPULAR TEXT-BOOKS on NURSING.

"Valuable little handbook."
*Nursing Notes.*

THE BURDETT SERIES.

"Can be strongly recommended to nurses."
*Treatment.*

Small Crown 8vo. Bound in cloth boards. Price 1/- each post free; or the Series complete 10/- post free.

Each Volume contains about 100 pages written by a competent authority on the subject treated.

No. 1.—PRACTICAL HINTS ON DISTRICT NURSING. By AMY HUGHES.

No. 2.—HOSPITAL AND PRIVATE NURSING. By S. E. ORME.

No. 3.—THE MIDWIVES' POCKET-BOOK. With Prefatory Chapter on the Midwives' Bill. By HONNOR MORTEN, Author of "The Nurse's Dictionary," &c., &c.

No. 4.—FEVERS AND INFECTIOUS DISEASES: their Nursing and Practical Management. By WILLIAM HARDING, M.D.Ed., M.R.C.P.Lond., Author of "Mental Nursing," &c.

No. 5.—NOTES ON PHARMACY AND DISPENSING FOR NURSES. By C. J. S. THOMPSON.

No. 7.—THE CARE OF CONSUMPTIVES. By W. H. DAW, M.R.C.S., L.R.C.P.Lond.

No. 8.—HOME NURSING OF SICK CHILDREN. By J. D. E. MORTIMER, M.B., F.R.C.S., L.S.A.

No. 9.—MENTAL NURSING. By WILLIAM HARDING, M.D.Ed., M.R.C.P.Lond.

No. 10.—SICK NURSING AT HOME. By Miss L. G. MOBERLY.

No. 11.—GYNÆCOLOGICAL NURSING. By G. A. HAWKINS-AMBLER, F.R.C.S., Author of "The Commonwealth of the Body."

No. 12.—SYLLABUS OF LECTURES TO NURSES. By ANDREW DAVIDSON, M.D.

*To be obtained of all Booksellers, or of*

The SCIENTIFIC PRESS, LTD., 28 & 29 Southampton Street, Strand, London, W.C.

### WORKS FOR NURSES.

**LESSONS ON MASSAGE.** By Mrs. MARGARET D. PALMER, Manager of the Massage Department and Instructor of Massage to the Nursing Staff of the London Hospital. Second Edition enlarged and revised; pp. xvi. + 262 with 118 Illustrations, plain and coloured. *Just Published.* Price 7/6 net.

**A MANUAL FOR STUDENTS OF MASSAGE.** By Miss M. A. ELLISON, L.O.S. Second Edition, edited by Miss G. MANLEY, pp. xii + 126. With 50 original Illustrations. *Just Published.* Price 3/6 net.

**A MANUAL FOR HOME NURSING.** By BERNARD MYERS, M.D., M.R.C.S., Surgeon and Lecturer to St. John's Ambulance Association. *Just Published.* Price 2/6 net.

**NOTES ON HOME NURSING.** By Miss D. M. GOLDIE, Lecturer on Nursing, Hygiene, &c., to the County Councils of London, Surrey and Middlesex. *Just Published.* Price 1/6 net.

**THE FEEDING AND HYGIENE OF INFANTS AND CHILDREN.** By JOHN McCAW, M.D., M.R.C.P., Physician to the Belfast Hospital for Children. *Just Published.* Price 2/6.

**DISEASES OF THE HAIR, INCLUDING GREYNESS, BALDNESS, &c., AND THEIR TREATMENT.** By DAVID WALSH, M.D., Western Hospital for Diseases of the Skin. Price 2/6 net.

**MENSTRUATION AND ITS DISORDERS.** By ARTHUR E. GILES, M.D., B.Sc., F.R.C.S., Surgeon Chelsea Hospital for Women. Price 2/6 net.

**MOVABLE ATLAS OF THE ANATOMY AND PHYSIOLOGY OF THE FEMALE GENERATIVE ORGANS AND OF PREGNANCY.** Second Edition. With Text. By ARTHUR E. GILES, M.D., F.R.C.S. Price 3/- net.

**MOVABLE ATLAS OF THE ANATOMY AND PHYSIOLOGY OF THE CHILD.** With Explanatory Text. By D'ARCY POWER, M.A.Oxon, M.B., F.R.C.S. Price 3/- net.

**BAILLIÈRE'S POPULAR MOVABLE ATLAS OF THE ANATOMY AND PHYSIOLOGY OF MAN.** Size 16 by 7½ inches. With Explanatory Text. By W. S. FURNEAUX, Special Science Teacher London School Board. Price 3/6 net.

London: BAILLIÈRE, TINDALL & COX, 8 Henrietta Street, Covent Garden.

